# Purification and Characterization of a CkTLP Protein from *Cynanchum komarovii* Seeds that Confers Antifungal Activity

**DOI:** 10.1371/journal.pone.0016930

**Published:** 2011-02-22

**Authors:** Qinghua Wang, Fuguang Li, Xue Zhang, Yongan Zhang, Yuxia Hou, Shengrui Zhang, Zhixia Wu

**Affiliations:** 1 College of Science, China Agricultural University, Beijing, China; 2 Cotton Research Institute, Chinese Academy of Agricultural Sciences, Anyang, Henan, China; University of Missouri-Kansas City, United States of America

## Abstract

**Background:**

*Cynanchum komarovii* Al Iljinski is a desert plant that has been used as analgesic, anthelminthic and antidiarrheal, but also as a herbal medicine to treat cholecystitis in people. We have found that the protein extractions from *C. komarovii* seeds have strong antifungal activity. There is strong interest to develop protein medication and antifungal pesticides from *C. komarovii* for pharmacological or other uses.

**Methodology/Principal Findings:**

An antifungal protein with sequence homology to thaumatin-like proteins (TLPs) was isolated from *C. komarovii* seeds and named CkTLP. The three-dimensional structure prediction of CkTLP indicated the protein has an acid cleft and a hydrophobic patch. The protein showed antifungal activity against fungal growth of *Verticillium dahliae*, *Fusarium oxysporum*, *Rhizoctonia solani*, *Botrytis cinerea* and *Valsa mali*. The full-length cDNA was cloned by RT-PCR and RACE-PCR according to the partial protein sequences obtained by nanoESI-MS/MS. The real-time PCR showed the transcription level of *CkTLP* had a significant increase under the stress of abscisic acid (ABA), salicylic acid (SA), methyl jasmonate (MeJA), NaCl and drought, which indicates that CkTLP may play an important role in response to abiotic stresses. Histochemical staining showed GUS activity in almost the whole plant, especially in cotyledons, trichomes and vascular tissues of primary root and inflorescences. The CkTLP protein was located in the extracellular space/cell wall by CkTLP::GFP fusion protein in transgenic Arabidopsis. Furthermore, over-expression of CkTLP significantly enhanced the resistance of Arabidopsis against *V. dahliae*.

**Conclusions/Significance:**

The results suggest that the CkTLP is a good candidate protein or gene for contributing to the development of disease-resistant crops.

## Introduction

During evolution, plants have formed a variety of effective defense mechanism against pathogen infection. Antimicrobial proteins compose a defense system in plants for resisting the infection of pathogenic microorganisms, and pathogenesis-related (PR) proteins are regarded as quick and powerful defense mechanisms against pathogens [1]. Based on their sequences and structures, PR proteins are classified into 17 families [2]. Members of one family, including PR-5 protein, osmotin-like protein, PR-like allergy, PR5-like protein kinase receptor and permatins like zeamatin, hordomatin and avematin [3], [4], are also called thaumatin-like proteins (TLPs) because they have sequence homology to the sweet protein-thaumatin from *Thaumatococcus daniellii* [5].

The TLPs are widely distributed in angiosperms [5] and gymnosperms [4], [6]. The TLPs after isolation and purification from plants show a broad-spectrum resistance and are able to resist multiple pathogens [4]. The antimicrobial molecular mechanism of TLPs is not fully clear. Studies have found that TLPs enhance the permeability of fungal cell membrane by forming a membrane hole, which enable water influx and cause rupture of the membrane [7]. In addition, TLP binding β-glucan has the activity of β-glucanase [8]; it can be used as a xylanase inhibitor [9]; it has the antifreeze activity and is involved in plant development, such as flower formation and fruit ripening [10], [11].

Most TLPs have relative molecular mass of 21–26 kDa and signature sequence of thaumatin family G-x-[GF]-x-C-x-T-[GA]-D-C-x(1,2)-[GQ]-x(2,3)-C [12]. The L-type TLPs include 16 conserved cysteine residues and S-type TLPs 10 conserved cysteine residues. The disulfide bridges formed by these cysteine residues have an important role in maintaining the protein stability and correct folding and preserving high activity under extreme temperature and pH conditions [13].

TLPs play an important role for genetically modified crops to improve the resistance to disease, drought and salt stress. For example, transgenic strawberry plants expressing thaumatin II gene have a higher level of resistance to *B. cinerea* [14]. Over-expression of TLP-1 from barley in transgenic wheat reduced in Fusarium head blight severity in green house evaluation [15]. Transgenic tobacco with a thaumatin gene delayed the disease symptoms caused by *Pythium aphanidermatum* and *R. solani*, and improved seed germination rate and seedling survival rate under salt and PEG-mediated drought stress [16]. Over-expression of an osmotin gene in transgenic tobacco showed higher tolerance to drought and salt [17], as well as an improvement of the salt tolerance in transgenic strawberry and the drought-resistance of transgenic cotton [18], [19].

*Cynanchum komarovii* Al Iljinski is a desert plant adapted to the dry and barren environment in the desertification process, and belonging to the Asclepiadaceae family. The plant has been used as analgesic, anthelminthic and antidiarrheal, and also as herbal medicine to treat cholecystitis in people. In addition, it can provide raw materials for producing pesticides in agriculture [20]. Although the chemical composition of *C. komarovii* such as total alkaloids showed antifungal activity, the antimicrobial proteins have huge potential in transgenic engineering.

In this paper, we report the isolation and characterization of an antifungal protein-TLP from *C. komarovii* seeds. We also show over-expression of the *CkTLP* gene in transgenic Arabidopsis performed or activated resistance against *V. dahliae*, which affects more than 400 plant types and limits the crop yield especially in cotton [21]. The results suggest that the CkTLP can significantly contribute to the development of disease-resistant crops.

## Results

### Purification of an antifungal protein from *C. komarovii* seeds

**Figure 1:**
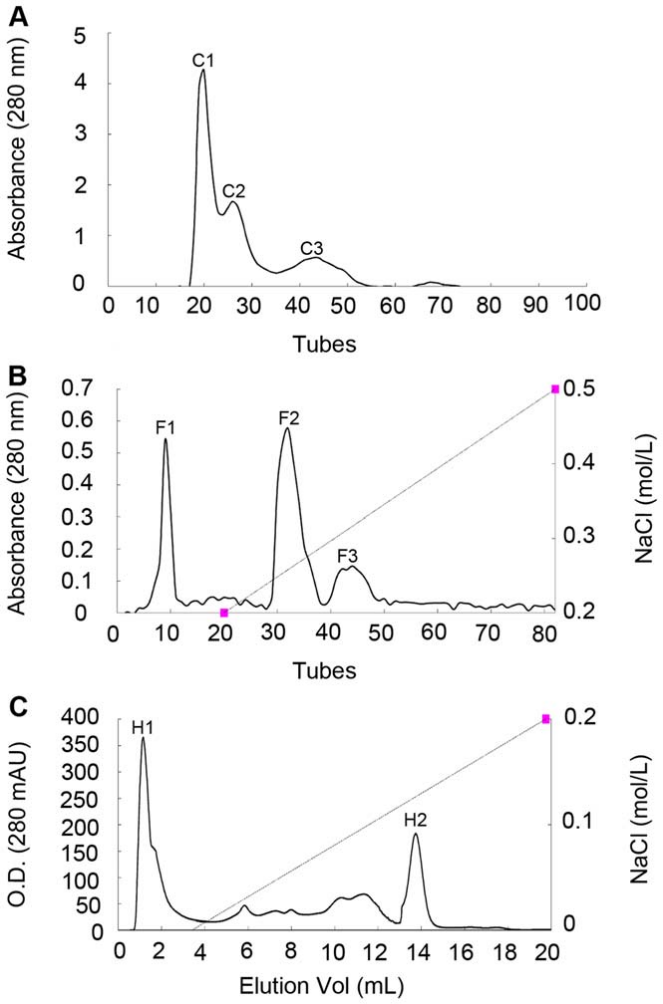
Isolation of antifungal proteins from *C.* *komarovii* seeds. **A.** The sample was loaded on Sephadex G-50 column (1.6×100 cm) with the flow rate of 18 mL/h. **B.** Antifungal fraction C1 was separated on cation-exchange SP-Sephorase Fast Flow column (1.6×30 cm) at 1 mL/min. **C.** Antifungal fraction F2 was loaded on FPLC-Resource S column.

**Figure 2:**
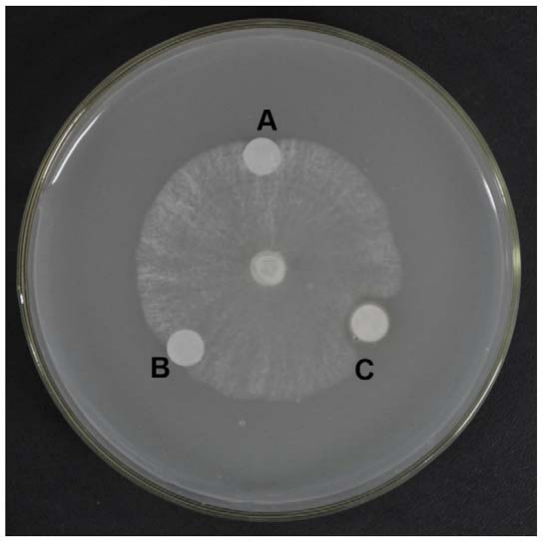
Antifungal activity of *C.* *komarovii* seeds proteins against *V.* *mali*. **A.** Phosphate buffer, pH 6.8. **B.** Boiled total protein. **C.** 10 µg purified CkTLP protein in 5 µL phosphate buffer, pH 6.8.

We found that the extractions from *C. komarovii* seeds have strong activity against several pathogenic fungi such as *V. dahliae*, *F. oxysporum*, *R. solani*, *B. cinerea* and *V. mali*. Extracted proteins precipitated with 40–60% ammonium sulfate were separated into three different fractions named C1, C2 and C3 by gel filtration chromatography in Sephadex G-50 (Figure 1A). The antifungal activities of fractions were assayed on the test fungus, *V. mali*. The fraction C1 with strong antifungal activity was collected and further fractionated. Three major fractions were obtained named F1, F2 and F3 by cation exchange chromatography (Figure 1B), and the antifungal activity was associated with F2 fraction. A partially purified concentrate of F2 was fractionated on Resource S column in FPLC (Figure 1C), to isolate antifungal protein H2. The isolated protein showed strong inhibition to the mycelial growth in *V. dahliae*, *F. oxysporum*, *R. solani*, *B. cinerea* and *V. mali*. The IC_50_ (half maximal inhibitory concentration) values of these fungi were 24, 20, 21, 11 and 3.0 µM respectively (Table 1). Antifungal activity of the purified protein against the test pathogenic fungus *V. mali* was displayed in Figure 2.

**Table 1:** Effect of CkTLP on fungi growth^1^.

Fungi species	Half maximal inhibitory concentration (µM)
*Verticillium dahliae*	24
*Fumrium oxysporum*	20
*Rhizoctonia solani*	21
*Botrytis cinerea*	11
*Valsa mali*	<3.0

1 Five doses of purified CkTLP were used for determination the half maximal inhibitory concentration (IC_50_) of antifungal activity. Buffer was used as a control.

### Characterization and identification of antifungal protein from *C. komarovii*

**Figure 3:**
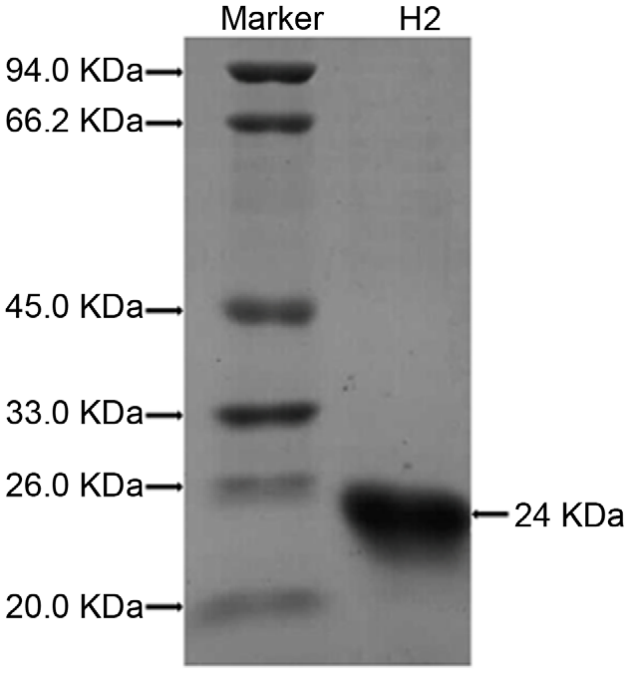
SDS-PAGE analysis of purified protein. SDS-PAGE analysis of H2, antifungal fraction on FPLC-Resource S column.

**Figure 4:**
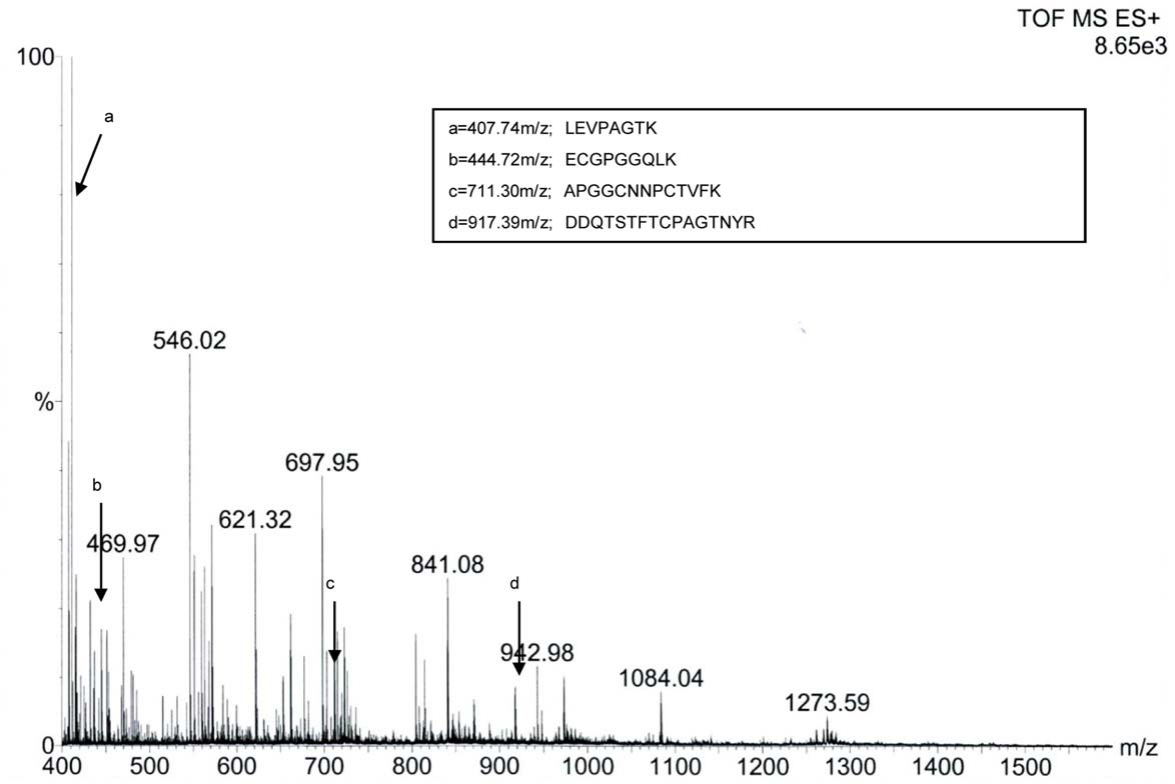
Electrospray ionization mass spectral analysis of antifungal protein. Antifungal protein of 24 kDa was submitted to nanoESI-MS/MS analysis and polypeptide fragments corresponding to peaks a, b, c and d are shown in the inset.

The analysis of antifungal protein profile showed that the H2 fraction contained only one protein with molecular mass about 24 kDa in SDS-PAGE (Figure 3). After in gel-trypsin digestion, the peptides were identified with nanoESI-MS/MS analysis and obtained the resulting pattern of fragmentation from MS. The four polypeptide sequence fragments were shown in the inset of Figure 4. The nanoESI-MS/MS analysis of each polypeptide revealed a long series of the complementary y- and b-type ion-fragments and their correspondence to the amino acid sequence on spectrum (Figure S1). The polypeptide sequences had high homology to PR-5 from *Daucus carota* (AY065642), *Solanum nigrum* (AF450276), *Arabidopsis thaliana* (CAA18495) and *Nicotiana tabacum* (CAA43854).

### Cloning of CkTLP cDNA

**Figure 5:**
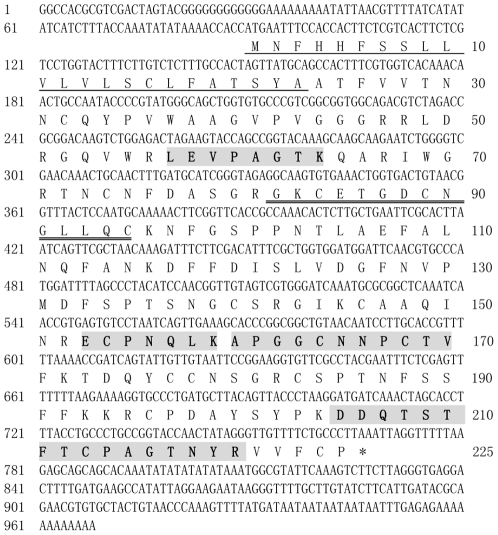
Nucleotide sequence of *CkTLP* and deduced amino acid sequence. The thaumatin family signature is marked by double underline. The signal peptide is underlined. The regions in shadow and bold indicate the four polypeptide fragments obtained from nanoESI-MS/MS of CkTLP.

**Figure 6:**
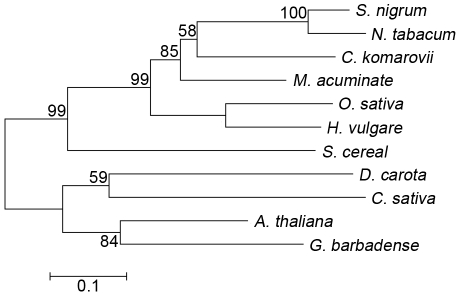
Phylogenetic tree of TLPs. The tree was constructed by the neighbor-joining method and bootstrap values are indicated at the branches. Amino acid sequences of TLPs come from *Cynanchum komarovii* (GU067481), *Daucus carota* (AY065642), *Musa acuminate* (GI: 88191901), *Solanum nigrum* (AF450276), *Secale cereal* (AF099671), *Arabidopsis thaliana* (CAA18495), *Nicotiana tabacum* (CAA43854), *Gossypium barbadense* (DQ912960), *Oryza sativa* (U77657), *Hordeum vulgare* (AAK55326), *Castanea sativa* (CAB62167).

Using RT-PCR and RACE methods, we cloned the full-length cDNA sequences of the *CkTLP* gene (GenBank accession number: GU067481). The entire coding region of CkTLP was analyzed and deduced (Figure 5). The deduced amino acid sequence confirmed an identical protein result with four matched polypeptide sequences from nanoESI-MS/MS. *CkTLP* contained an open reading frame (ORF) with 675 bp, encoding a protein of 225 amino acids (aa), p*I* 8.6, molecular weight about 24 kDa, including 16 cysteine residues. In addition, the deduced protein has a thaumatin family signature GxCxTGDCxGx(2,3)C at 82 aa, an N-glycosylation site at 187 aa and a typical feature of signal peptide at 1-23 aa. In the BLAST search, CkTLP shared high identity with TLPs from *Musa acuminate* (76%) and *S. nigrum* (61%). Phylogenetic analysis of TLPs differentiated five main branches (Figure 6), in which CkTLP was grouped with *M. acuminate* (GI: 88191901), *S. nigrum* (AF450276) and *N. tabacum* (CAA43854). The phylogeny suggests that they may have similar features and functions [22], [23], [24].

### *CkTLP* transcript levels are regulated by stresses

**Figure 7:**
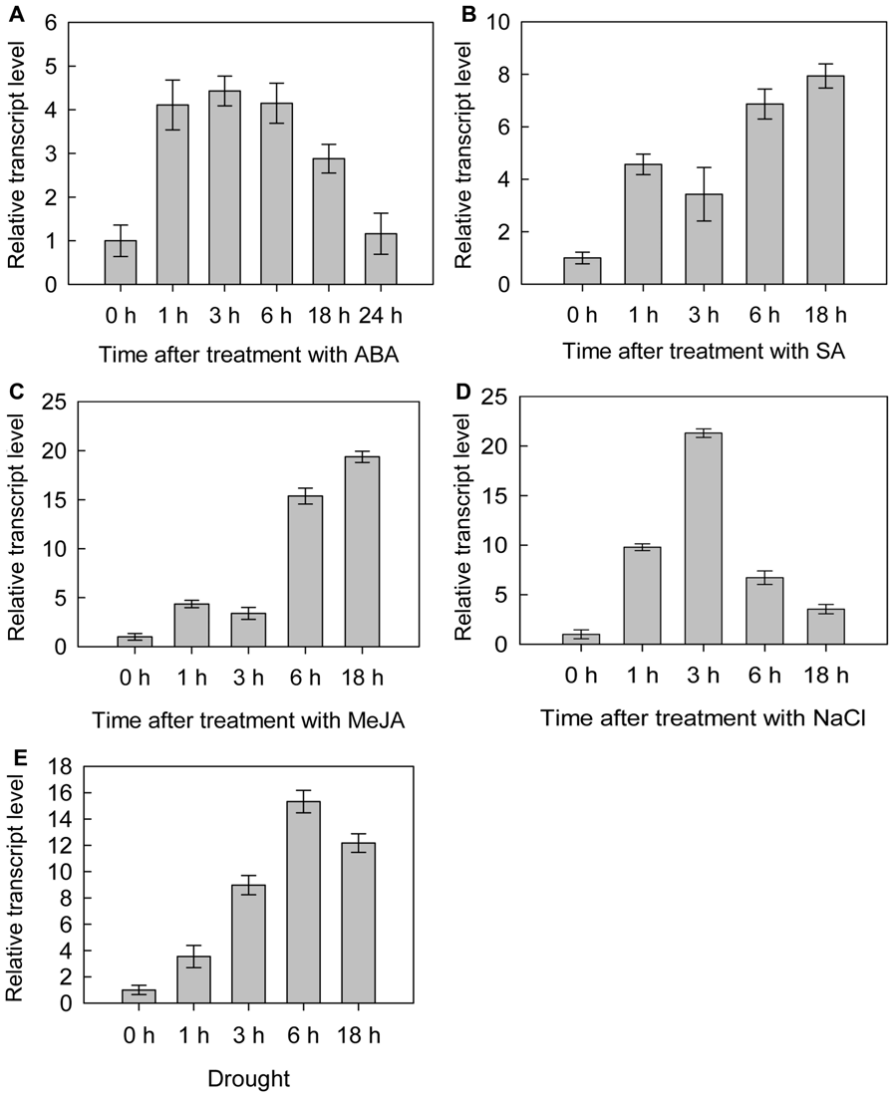
Relative mRNA abundance of *CkTLP* at various time post-treatment. **A.** ABA **B.** SA **C.** MeJA **D.** NaCl **E.** Drought. Results are expressed as means±SD (n = 3).

To study the transcript levels of *CkTLP* in response to abiotic stresses, *C. komarovii* seedlings were treated with different chemical inductions. In ABA treatment, the transcription of *CkTLP* was up-regulated at the early stage, until it reached the maximum, 4.43±0.34-fold over basal activity at 3 h post-treatment, and was down-regulated at the late stage and decreased rapidly at 24 h (Figure 7A). The transcription of *CkTLP* in SA treatment was up-regulated and reached 4.57±0.39-fold over basal level at 1 h time point, but the level decreased to 3.43±1.02-fold at 3 h. Then, the transcription of *CkTLP* continued to increase for 6 h-18 h, and achieved the maximum of 7.94±0.89-fold at 18 h (Figure 7B). The accumulation of *CkTLP* mRNA in response to MeJA increased to 4.35±0.37-fold at 1 h, but descended slightly at 3 h, then climbed to 19.38±0.57-fold of the basal level till 18 h (Figure 7C). The *CkTLP* mRNA accumulation in NaCl (300 mM) increased to 9.79±0.34-fold at 1 h time point, remained at 21.31±0.43-fold high levels for 3 h, and rapidly decreased during 6–18 h after treatment (Figure 7D). Drought of *C. komarovii* seedlings affected the *CkTLP* transcription (Figure 7E). The *CkTLP* continued to increase to 15.33±0.85-fold till 6 h, but a slightly declined at 18 h. These transcript profiles indicate that *CkTLP* is responsive to different stresses.

### GUS histochemical analysis

**Figure 8:**
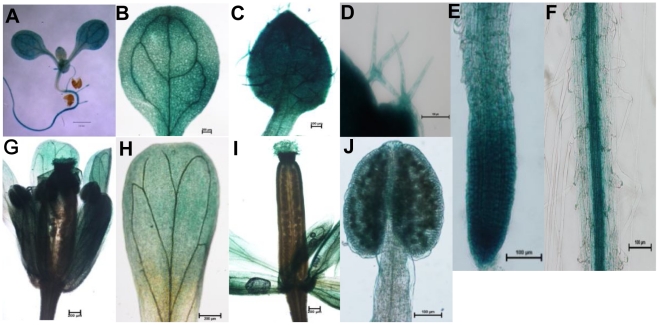
Histochemical staining of transgenic Arabidopsis. **A.**10-day-old seedling. **B.** Cotyledon. **C.** Leaf. **D.** Trichomes. **E.** Root tip. **F.** Root. **G.** Flower. **H.** Petal. **I.** Stigma. **J.** Anther. Scale for bars shown in Figures: 1 mm for A, 0.2 mm for B, G, H and I, 0.1 mm for C, D, E, F and J.

In order to assess CkTLP function in more details, the protein was expressed in Arabidopsis. Histochemical staining of T2 transgenic 10-day-old seedlings revealed that the CkTLP activity was shown in the whole plant (Figure 8A), containing cotyledon (Figure 8B), leaf (Figure 8C), trichomes (Figure 8D), root tip (Figure 8E), and root (Figure 8F) In addition, the GUS activity was also presented in the flower (Figure 8G) including petal (Figure 8H), stigma and anther (Figure 8I and J).

### Subcellular localization

**Figure 9:**
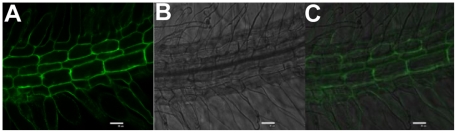
Subcellular localization of CkTLP::GFP in Arabidopsis. **A.** GFP. **B.** Bright-field. **C.** Overlap. Scale bars represent 20 µm.

In order to analyze the subcellular localization of CkTLP, we first used the ProComp version 9.0 program and predicted that CkTLP would be located in the extracellular space (score = 9.5). Then, the subcellular localization of the CkTLP within plant cell was assessed by the fusion protein CkTLP::GFP. Bright fluorescence was observed in extracellular space of seedling root cells by Confocal Laser Scanning Microscopy (Figure 9). In order to differentiate between the cytoplasm membrane and cell wall location, the seedlings were treated with 30% sucrose solution for about 2 h. No difference of fluorescence in the plasmolyzed cell was observed, suggesting that CkTLP is located in the extracellular space or cell wall.

### Disease resistance of CkTLP in transgenic Arabidopsis

**Figure 10:**
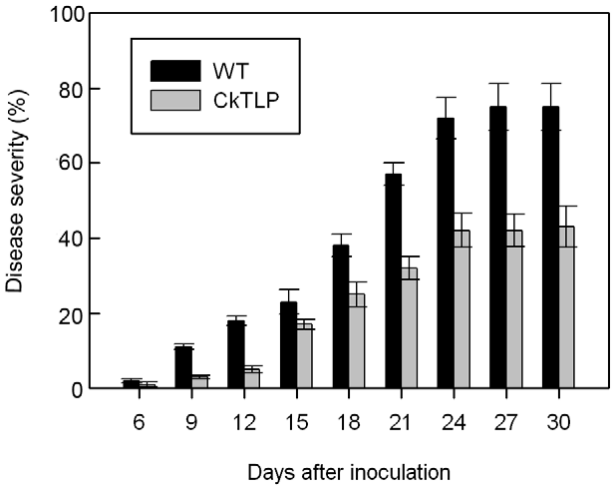
Disease severity of wild-type and transgenic Arabidopsis after inoculation by *V.* *dahliae*. The vertical bars indicate the standard errors (P<0.05).

Wild-type and transgenic Arabidopsis were inoculated with *V. dahliae* after growing for 3 weeks in the growth chamber. Disease severity (percentage of leaves with wilt symptom) was recorded starting at 6 days post-inoculation (dpi) and until 30 dpi (Figure 10). The wild-type plants showed more prominent symptoms than transgenic plants, and the disease incidence was 75% at 30 dpi, in the transgenic plants the rate was 43%. These results implicate the CkTLP protein is capable of protecting plants against fungal diseases.

### Analysis of the predicted three-dimensional structure of the CkTLP protein

**Figure 11:**
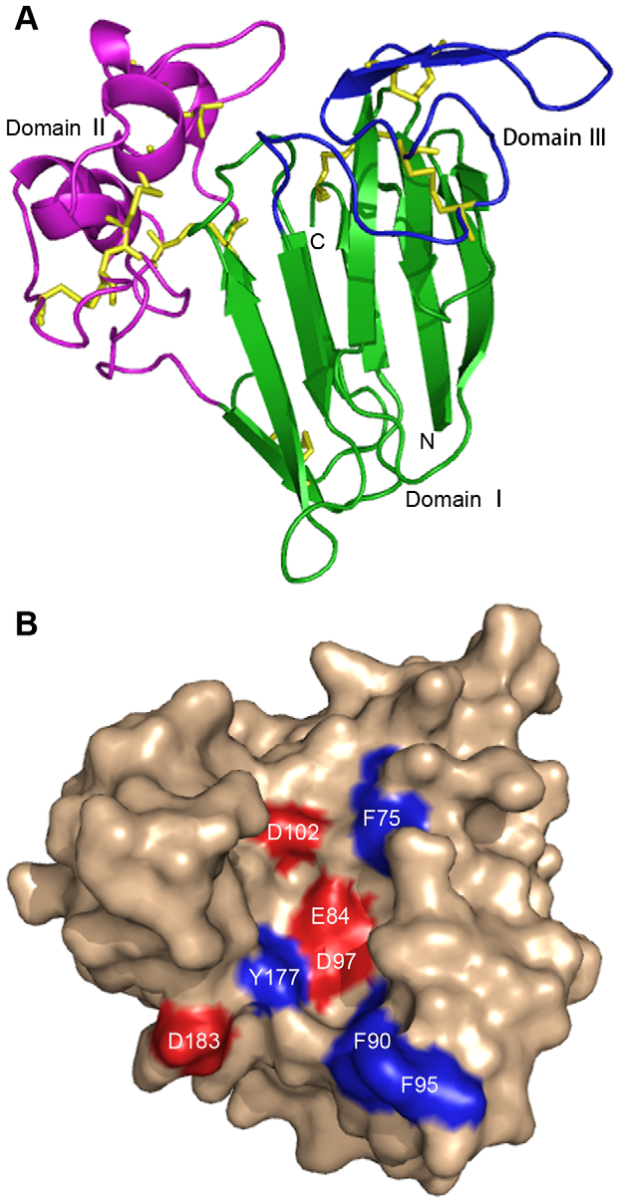
Predicted three-dimensional structure of CkTLP. **A.** A ribbon diagram of CkTLP showing the three domains in green (I), magenta (II) and blue (III). The eight disulfide bridges in structure are shown in yellow. The cleft region lies at the interface of domains I and II. **B.** Representation of the molecular surface of CkTLP. The acidic cleft is colored red. The surface hydrophobic residues are displayed in blue.

To understand the mechanism of CkTLP antifungal activity, we predicted the three-dimensional structure by computer modeling (Figure 11).The model was built using the template protein from *M. acuminate* which had 76% sequence identity with CkTLP by SWISS-MODEL server. The CkTLP model had three structural domains (Figure 11A), domain I was built a β-sandwich by two β-sheets comprising six and five antiparallel strands forming the front and back sheet, respectively; domain II was composed of an extended α-helix and four shorter α-helices, and domain III formed a hairpin segment of two short strands of β-sheets linked to an extended loop. The model showed CkTLP formed 8 disulfide bridges by 16 cysteine residues, 9 to 201, 51 to 61, 72 to 66, 117 to 190, 123 to 173, 131 to 141,145 to 154 and 155 to 160, which was important for forming the backbone of the protein. Domain I and II formed a central cleft by four inside acidic residues (E84, D97, D102 and D183) and four superficial hydrophobic residues (F75, F90, F95 and Y177) ( Figure 11B). The similar electronegative central cleft have explained the antifungal activity in *M. acuminate* [22]. It suggests that the predicted electronegative cleft from CkTLP may be an essential feature for the observed antifungal activity.

## Discussion

TLPs have been purified from a variety of monocotyledonous and dicotyledonous plants. They are believed to be effective PR proteins that function to inhibit the growth of microorganisms. In this study, a new antifungal protein (CkTLP) was isolated from *C. komarovii* seeds with its purification guided by antifungal activity, which belonged to the thaumatin-like family. The TLP superfamily contains at least 31 genes in *Oryza sativa*, 28 in *Arabidopsis thaliana*, 13 in white spruce (*Picea glauca* Moench), 10 in western white pine (*Pinus monticola* Dougl.ex D. Don), 6 in moss, based on available plant genomes and EST databases [4]. We presumed that *C*. *komarovii* may have additional *CkTLP* homologs that are worthy of further studies.

The TLPs of plants have a higher transcription level under both biotic and abiotic stresses. For instance, the transcription level of *TLP* showed marked increase after treatment with ABA and elicitors (chitosan, β-glucan and cell-wall fragments of *F*. *oxysporum*) in the apoplast of wheat [25]. TLP accumulated in wheat via jasmonic acid (JA) and SA signaling against *Stagonospora nodorum* [26]. Although expression of the *dcTLP* from *Daucus carota* was not affected by ABA, SA and JA directly, it had a high level specific to drought stress in the embryogenic calli, seedlings, and mature plants [27]. ABA is considered to be able to modulate gene expression under osmotic-related stress such as freezing, drought and salt, but not all osmotic-induced genes appear to ABA dependent. In ABA treatment, the transcript of *CkTLP* was up-regulated to the maximum 4.43±0.34-fold at 3 h post-treatment (Figure 7A). In NaCl (300 mM), it remained at 21.31±0.43-fold high levels for 3 h (Figure 7D), and in drought, the accumulation of *CkTLP* increase to 15.33±0.85-fold at 6 h (Figure 7E). These results indicated that CkTLP is involved in *C. komarovii* response to osmotic stresses. Furthermore, we found the transcription of *CkTLP* in SA and MeJA treatment was up-regulated respectively (Figure 7B and C). It indicated that exogenous SA and MeJA can induce the expression of *CkTLP* and may establish systemic acquired resistance (SAR) against the pathogens. These results suggest that the CkTLP may be involved in host defence to abiotic stresses.

The GUS-fusion gene was driven by CaMV35S promoter which could make genes constitutively express in each organ of plants, without temporal-spacial speciality and affected by any factors [28]. In this research, *CkTLP-GUS* was expressed in both vegetative and procreative organs, according with the activity of CaMV35S promoter. However, the GUS intensity was stronger in cotyledons, vascular tissues of primary root and inflorescences than other organs (Figure 8). Surprisingly, real-time PCR analysis on the *CkTLP* expression patterns of *C. komarovii* displayed more abundant mRNA in seed, flower, cotyledon and root than stem and leaf, which was consistent with GUS activity except seed (Figure S2). Although the reason for the coincidence was unclear, *CkTLP* had different expression constitutively in an organ-referred or development-dependent pattern as concluded in plant TLPs [4]. For example, *TLP* from elderberry (*Sambucus nigra*) showed a fruit-specific expression [29]; *TLP* of Japanese pear (*Pyrus serotina* Burm.f.) expressed in the pistils, anthers at low level, and nothing in other floral organs and leaves [30]; in developing barley and oats seeds, *TLP* expression initially occurs in the ovary wall then switches to the aleurone [31]; and TLP of cotton showed high levels of expression in fiber cell involved in secondary cell wall development [32]. It was interesting to note that seeds of *C*. *komarovii* had the highest transcription levels of CkTLP (Figure S2), which further indicate the expression pattern is preferentially expressed in seeds.

Many TLPs have been isolated from plants and possess antifungal or antibacterial properties. For instance, the VvTLP-1 from grapevine inhibited the hyphae growth and spore germination of *Elsinoe ampelina* [33]. The TLP from chestnut (*Castanea sativa* Mill) exhibited antifungal activity against *Trichoderma viride* and *F. oxysporum* and had a synergistic effect with endochitinase from the chestnut cotyledon [34]. The CkTLP protein from *C. komarovii* displayed an obvious inhibitory effect against *V. mali*, *B. cinerea*, *R. solani*, *F. oxysporum* and *V. dahliae* (Table 1). Compared to TLPs from French bean legumes [35] and kiwi fruits [36], the CkTLPs in vitro showed more potent inhibitory activity on *F. oxysporum* and *R. solani*, yet lower activity against *F. oxysporum* than TLP of Kweilin chestnut [37], as well as lower inhibition on *V. dahliae* than ATLP-3 of Arabidopsis by bacterially expressed protein [38]. Over-expression of the CkTLP in transgenic Arabidopsis led to increased resistance of these plants against *V. dahliae* (Figure 10). These results suggested that CkTLP was a novel, antifungal protein and potential application in transgenic plants. In addition, the CkTLP was located in the extracellular space or cell wall (Figure 9), which may be more effective against the fungus invasion and spread, since fungal penetration through plant cell walls and cell membranes is an important pathway to suppress plant defense system. So far, the TLPs are considered to be able to insert into plasma membrane and form pores [7], though the crystal structure of zeamatin suggested it is unlikely to be involved directly in forming pores in the plasma membrane [39]. The antimicrobial molecular mechanism of CkTLP should be further investigated.

In addition, the three-dimensional structure of CkTLP was simulated (Figure 11). The presence of surface negative electrostatic cleft resulting from four acidic residues was considered to determine the character of antifungal activity [22], [23]. The conclusion supports the prediction that the antifungal activity of CkTLP is due to the functional patch composed of E83, D97, D102 and D183. Furthermore, CkTLP had an allergen domain at 106-146 aa, whose function need to be determined in the future.

In summary, we have purified a TLP from *C. komarovii* seeds and identified the character of the *CkTLP* gene and function in transgenic Arabidopsis. The three-dimensional structure by computer simulation can help us understand the mechanism of CkTLP antifungal activity. Although the mechanism of antifungal activity awaits further studies, CkTLP can provide a good candidate protein for protecting crops against some pathogenic fungi.

## Materials and Methods

### Plant and fungal growth conditions and treatments

*C. komarovii* seeds were provided by the lab of molecular biology of China Agricultural University. The seeds were planted in a mixture of soil and vermiculite (2∶1, w/w) and seedlings were cultivated under a photoperiod of 16 h light/8 h dark, day and night temperature of 25°C and 22°C in the greenhouse of China Agricultural University. After three weeks, the seedlings were carefully extracted from pots and the soil washed off, then rapidly immersed in liquid nitrogen, and stored at −80°C.

The seeds of Arabidopsis (ecotype Columbia) were provided by the lab of molecular biology of China Agricultural University. Seeds were treated with 4% NaClO solution, 75% ethanol and sterilized water in turn for surface sterilization, then sowed on MS culture medium (1×MS salts, 1×MS vitamins, 3% sucrose, 1% agar, pH 5.7). After iarovization for 2 d at 4°C, the seeds were cultured in a growth chamber under 16 h light at 23°C and 8 h dark at 18°C, with 80% relative humidity. After 10 d, seedlings of Arabidopsis were transplanted in pots with mixture of soil and vermiculite (1∶1, w/w), under the same growth conditions.

For the treatment experiments, the seedlings of *C. komarovii* were carefully removed from soil and blotted up water then placed into the solutions with ABA (100 µM), SA (1 mM), MeJA (100 µM) and NaCl (300 mM), using three repeated trials. Control samples were treated with water. The seedlings were incubated in the same temperature and moisture. Samples were taken at 0, 1, 3, 6 and 18 h after the stress treatments with ABA, SA, MeJA and NaCl, respectively. For drought treatment, the seedlings were treatment with 20% (w/v) polyethylene glycol (PEG6000) at 25°C for 0, 1, 3, 6 and 18 h to determine the transcript levels.

Fungi were provided by the Department of Plant Protection, China Agricultural University. The fungal species *Verticillium dahliae* Kleb, *Fusarium oxysporum* f. sp. *vasinfectum*, *Rhizoctonia solani* Kühn, *Botrytis cinerea* f. sp. *cucumerinum* and *Valsa mali* Miyabe et Yamada were grown in potato dextrose agar (PDA) plates at 25°C. The antifungal activity assay in vitro and determination of IC_50_ was performed as previously described [40] and partly modified. For the assay for antifungal activity, the fungi were grown in PDA plates and inoculated at 25°C for 48 h. Sterile filter paper discs (0.6 cm in diameter) were placed at a distance of 0.5 cm from the edge of the mycelial colony. The samples (proteins) and control (phosphate buffer (PBS), pH 6.8) were added to the paper disks. The development of mycelium was taken as a measure of growth. To determine the IC_50_ for the pure protein, five different doses (0.3, 1.2, 4.8, 15.2 and 60.8 μM) of the antifungal protein and buffer (PBS, pH 6.8) were added in PDA plates at 45°C, and then a small amount of mycelia (0.5 cm in diameter) were placed on the surface of the agar. After incubation at 25°C for 48 h, the area of the mycelial colony and the fungal growth inhibition was measured. The determination of concentration of antifungal protein required for a 50% inhibition of fungal growth. The conidial suspensions of *V. dahliae* were prepared from a culture grown for 5 days at 25°C in Czapek liquid medium (NaNO_3_ 2 g, K_2_HPO_4_ 1 g, MgSO_4_^.^7H_2_O 0.5 g, KCl 0.5 g, FeSO_4_ 0.01 g, sucrose 30 g, distilled water 1 L, pH 7.2). All fungi were used for detection of antifungal proteins in vivo. The analysis of resistance in transgenic Arabidopsis was performed on the conidial suspensions (5×10^6^ conidia/mL) of *V. dahliae*.

### Purification of antifungal proteins

The method of purification of the antifungal proteins from *C. komarovii* seeds was performed basically according to Ho et al. [40]. 100 g seeds were milled and fully homogenized with 2–3 volumes extraction buffer (50 mM Tris-HCl, 10 mM KCl, 1 mM EDTA,1 mM phenylmethylsulfonyl fluoride (PMSF), 5 mM iodoacetic acid, pH 7.5), at 4°C. After overnight incubation, the homogenate was filtered through four layers of gauze to remove un-dissolved materials and the filtrate was centrifuged at 12,000 rpm for 30 min at 4°C. The supernatant was precipitated in 40% saturated ammonium sulfate solution. After centrifugation, the resulting supernatant was added to 60% saturated ammonium sulfate solution, and precipitated for 4 h on ice. The crude proteins were separated by centrifugation (12,000 rpm, 30 min at 4°C) and re-suspended in 40 mL buffer (50 mM Tris-HCl, pH 7.5) and dialyzed extensively against the buffer using 1-kDa-cutoff dialysis membrane. The dialyzed solution was condensed by freeze drying and submitted to chromatographic methods. A gel filtration Sephadex G-50 (GE Healthcare, NJ, USA) column (1.6×100 cm) was used for further separation of proteins from the dialyzed solution. The column was equilibrated with 50 mM Tris-HCl (pH 7.5) containing 0.1 M NaCl. The fraction that showed high inhibitory activity was collected and further purified by SP-Sepharose Fast Flow ( GE Healthcare, NJ, USA) cation-exchange column (1.6×30 cm). The column was equilibrated and initially eluted with 10 mM ammonium acetate buffer (pH 4.3). Adsorbed protein was eluted using a linear eluant strength gradient (0.2–0.5 M NaCl) in equilibration buffer. The resulting sample with antifungal activity was finally purified on AKTA FPLC Resource S column (GE Healthcare, NJ, USA). The chromatography was equilibrated with 20 mM PBS (pH 6.8) and adsorbed protein was eluted in linear gradient using NaCl from 0 to 0.2 M in the equilibration buffer. The identification and purity of the samples were confirmed by SDS-PAGE (12% gel).

### Partial amino acid sequencing

The antifungal protein was identified by nanoESI-MS/MS (Micromass, Manchester, UK). Protein sequences homology searches were applied using BLAST compared the protein sequence with known proteins or translated ORFs of expressed sequence tags (ESTs) in the database at NCBI.

### Cloning of *CkTLP*

Total RNA of *C*. *komarovii* seedlings were obtained by an extraction kit (Promega WI, USA). To obtain cDNA encoding CkTLP, we designed two degenerate primers: F- ACCAACTGCAACTTCGHTGN; and R-TTGGTACCRSYAGGGCAWGT, which was based on the determined proteins sequences TNCNFDA and TCPAGTN by nanoESI-MS/MS. The first-strand cDNA was obtained from total RNA using the reversed transcription kit (Takara, Shiga, Japan). 3′ RACE was performed using RT-PCR product as template with two nested gene-specific sense primers, GPS1 (5′GCCCTACATCCAACGGTTGTAG3′) and GPS2 (5′CAACCGTGAGTGTCCTAATCAG3′), following the specification of 3′RACE kit (Takara, Shiga, Japan). 5′RACE cDNA was obtained with 5′RACE kit according to the manufacturer's instructions (Invitrogen, CA, USA) and first-strand cDNA synthesis was performed with the antisense primer GPS3 (5′CTGATTAGGACACTCACGGTTGA3′). The tailed cDNA was used to as template for amplification with the adapter primer and two nested primers GPS4 (5′CGCATTTGATCCCACGACTAC3′) and GPS5 (5′GGCACGTTGAATCCATCCACCA3′). The reaction of RT-PCR started at 94°C for 5 min, and following cycle was repeated 33 times: 94°C for 30 s, 52–55°C for 30 s, 72°C for 1 min, and final extension went on at 72°C for 10 min. The reaction products were purified and cloned into pGEM-T vector (Promega, WI, USA) for sequencing.

### Sequence and phylogenetic analysis

The detected amino acid sequence was carried out using DNAMAN tool. Sequence features, such as signal peptide, p*I* and molecular mass were evaluated using protein analysis tools (http://expasy.org/tools). TLPs sequences were selected from NCBI. The mature protein sequences were aligned with Cluster X version 2.0 and gaps were removed from the alignment. The phylogenetic tree of those alignments was calculated by the neighbor-joining method using MEGA 4 program, and bootstrap values from 1000 replicates indicated at the branches.

### Real-time PCR

Total RNA was isolated from stored *C*. *komarovii* seedlings after different treatments using a RNA extraction kit (Promega, Madison, WI, USA). 2 µg total RNA was reverse transcribed to first-strand cDNA using the High Capacity RNA-to-cDNA kit (Applied Biosystems, Foster City, CA, USA) according to manufacturer specifications. Diluted cDNA was used as template in each well for the quantitative real-time PCR analysis. The unique primers of *CkTLP* were designed with TLP-F (5′CGACATTTCGCTGGTGGATG3′) and TLP-R (5′CTGTAAGCATCAGGGCACCT3′). The endogenous control was *EF-1-*α (GenBank accession number: HQ849463) from *C. komarovii* and identified with the sense primer EF1α-F (5′ TGCATCCAACTCGAAGGATG 3′) and antisense primer EF1α-R: (5′ CCTTACCAGATCGTCTGTCT 3′). The real-time PCR was performed by 20 μL reaction mixture in 96-well plate with ABI7500 thermocycler (Applied Biosystems, Foster city, CA, USA), using Power SYBR Green PCR Master Mix (Applied Biosystems, Foster city, CA, USA). The PCR solution included 2 µL (20 ng) diluted cDNA, 10 µL SYBR Green Master Mix, 0.1 µmol forward and reverse primers, and final volume of 20 µL. The following condition was used in real-time PCR: 95°C denaturation for 10 min, 40 cycles of 95°C for 15 s, 58°C for 20 s, and 72°C for 30 s. Before proceeding with real-time PCR, we routinely verified that the primers of *CkTLP* and *EF-1-α* gene had a similar slop with high correlation coefficients by constructing standard curve (R^2^ = 0.96 and R^2^ = 0.95 respectively). The threshold cycle (CT) values of the triplicate real-time PCRs were averaged and the fold changes of transcription levels of target gene (*CkTLP*) relative to the reference gene (*EF-1-a*) was analyzed by the comparative CT (2^−ΔΔCT^) method, where ΔΔC_T_ =  (C_T_ target−C_T_ reference)_ Sample X_− (C_T_ target−C_T_ reference)_ Sample 1_. Sample 1 of *CkTLP* gene was calibrator sample without any treatments, whereas sample X was treated by different stresses. All experiments were repeated three times for cDNA prepared from three batches of plants. Statistical analysis of real-time PCR date and SD (Standard Deviation) values were performed as previously described by Livak et al. [41].

### Vector construction and plant transformation

The coding region of CkTLP was amplified by RT-PCR, using primers (5′ACCAAGATCTTATGAATTTCCACCACTTC3′ and 5′ACAAAGATCTGGGCAGAAAACAACAATA3′) to generate a *Bgl* II restriction site (underlined) at the 5′ end and 3′ end. The resulting DNA fragment was sub-cloned into the pCAMBIA1304 vector containing a hygromycin phosphotransferase (hph) gene, a fusion of β-glucuronidase (GUS) and green fluorescent protein (GFP) gene under the control of the CaMV35S promoter. To determine the function of CkTLP in Arabidopsis, the constructed vector with CkTLP was introduced into Arabidopsis (ecotype Colombia) via *Agrobacterium tumefaciens* (GV3101) transformation [42]. Transgenic Arabidopsis seeds were selected by hygromycin (25 mg/L) and the survived seedlings were confirmed by PCR. The PCR using genomic DNA of transgenic Arabidopsis leaves as template was carried out with the vector specific primers: 1304-F (5′GACCCTTCCTCTATATAAG3′) and 1304-R (5′GGACAACTCCATGAAAAG3′).

### GUS activity assay

For the histochemical analysis of GUS, T2 transgenic Arabidopsis were stained in the GUS solution (100 mM PBS), pH 7.0, 0.25 mM K_3_Fe(CN)_6_, 0.25 mM K_4_Fe(CN)_6_, 1 mM EDTA,1 mg/mL X-Gluc, 0.1% Triton X-100, 1-2 dips Tween 20). The samples were incubated in GUS staining solution at 37°C overnight. After staining, tissues were cleaned in 70% ethanol until no chlorophyll could be observed. GUS activity was examined under Stereomicroscope (Olympus, Tokyo, Japan) and Inverted Microscope (Nikon Ti, Tokyo, Japan).

### Subcellular localization

Intracellular localization of CkTLP was determined by CkTLP::GFP fusion protein in transgenic Arabidopsis. The roots of 10-day-old transgenic seedlings were detected by Confocal Laser Scanning Microscopy (Nikon C1, Tokyo, Japan). Excitation light at 488 and 543 nm was attenuated to 50% transmittance. Detectors were set at 610 nm for chlorophyll and 530 nm for GFP fluorescence.

### Analysis on transgenic Arabidopsis

To test transgenic Arabidopsis resistance against infection of *V*. *dahliae*, the bioassay was performed. The transgenic Arabidopsis seeds were sowed in sterilized soil for 3 weeks, then the seedling roots were drenched in 1 mL conidial suspensions (5×10^6^ conidia/mL) following the method of Tjamos et al. [43], and control seedlings were mock inoculated in distilled water. Disease severity was detected by the percentage of diseased leaves number over the total leaves per plant and periodically recorded for 30 days after inoculation. The experiment was repeated three times with 15 plants per treatment.

### Structural modeling of CkTLP

The three-dimensional structure of CkTLP was carried out by the SWISS-MODEL server (http://swissmodel.expasy.org/), and structure template was TLP of *M. acuminate* whose PDB code is 1Z3Q [22].

## Supporting Information

Figure S1 **nanoESI-MS/MS spectrums analysis of four polypeptide fragments from CkTLP protein.** **A.** nanoESI-MS/MS spectrum of the [M+H] ^2+^ ion (m/z 407.74). The mass differences of the consecutive y_n_ ions: m/z 814.49(y_8_), m/z 701.41(y_7_), m/z 572.36 (y_6_), m/z 473.29 (y_5_), m/z 376.24(y_4_), m/z 305.20 (y_3_), m/z 248.17 (y_2_) and m/z 147.24 (y_1_) and their correspondence to the amino acid sequence at the top of spectrum are shown. **B.** nanoESI-MS/MS spectrum of the [M+H] ^2+^ ion (m/z 444.72). The mass difference of the consecutive y_n_ ions: m/z 888.44(y_9_), m/z 759.40(y_8_), m/z 656.37 (y_7_), m/z 599.37 (y_6_), m/z 502.32 (y_5_), m/z 445.14(y_4_), m/z 388.28 (y_3_), m/z 260.21 (y_2_) and m/z 147.08 (y_1_) and their correspondence to the amino acid sequence at the top of spectrum are shown. **C.** nanoESI-MS/MS spectrum of the [M+H] ^2+^ ion (m/z 711.30). The mass difference of the consecutive y_n_ ions: m/z 1421.66(y_13_), m/z 1350.62 (y_12_), m/z 1253.57 (y_11_), m/z 1196.56(y_10_), m/z 1139.53(y_9_), m/z 979.51 (y_8_), m/z 865.47 (y_7_), m/z 751.41 (y_6_), m/z 654.34 (y_5_), m/z 494.32(y_4_), m/z 393.26 (y_3_), m/z 294.19 (y_2_) and m/z 147.12 (y_1_) and their correspondence to the amino acid sequence at the top of spectrum are shown. **D.** nanoESI-MS/MS spectrum of the [M+H] ^2+^ ion (m/z 917.39). The mass difference of the consecutive y_n_ ions: m/z 1833.78 (y_16_), m/z 1718.76 (y_15_), m/z 1603.76(y_14_), m/z 1475.70 (y_13_), m/z 1374.68(y_12_), m/z 1287.63 (y_11_), m/z 1186.59 (y_10_), m/z 1039.51 (y_9_), m/z 938.47 (y_8_), m/z 778.43 (y_7_), m/z 681.41 (y_6_), m/z 610.32 (y_5_), m/z 553.31(y_4_), m/z 452.25 (y_3_), m/z 338.31 (y_2_) and m/z 175.13(y_1_) and their correspondence to the amino acid sequence at the top of spectrum are shown.
(TIF)

Figure S2 **Real-time PCR analysis of** ***CkTLP*** **relative transcript level in different tissues of** ***C. komarovii*** **.** Total RNA were extracted from roots, stems, leaves of 3-week-old plants, cotyledons of 1-week-old seedlings and mature seeds. The CT values of CkTLP obtained from real-time PCR were normalized against those of EF1-α (yielding ΔCT±SD with three biological and three technical replicates). The relative transcript level of CkTLP was calculated using the formula X_fold_ = 2^−ΔΔCT^ with leaf used as reference condition. Date represent mean±SD (n = 3).
(TIF)
