# Supplementary figures and images for: Purification and Characterization of a CkTLP Protein from Cynanchum komarovii Seeds that Confers Antifungal Activity
Source: PLoS One. 2011 Feb 22;6(2):e16930. doi: 10.1371/journal.pone.0016930 (PMC3043079; doi:10.1371/journal.pone.0016930)

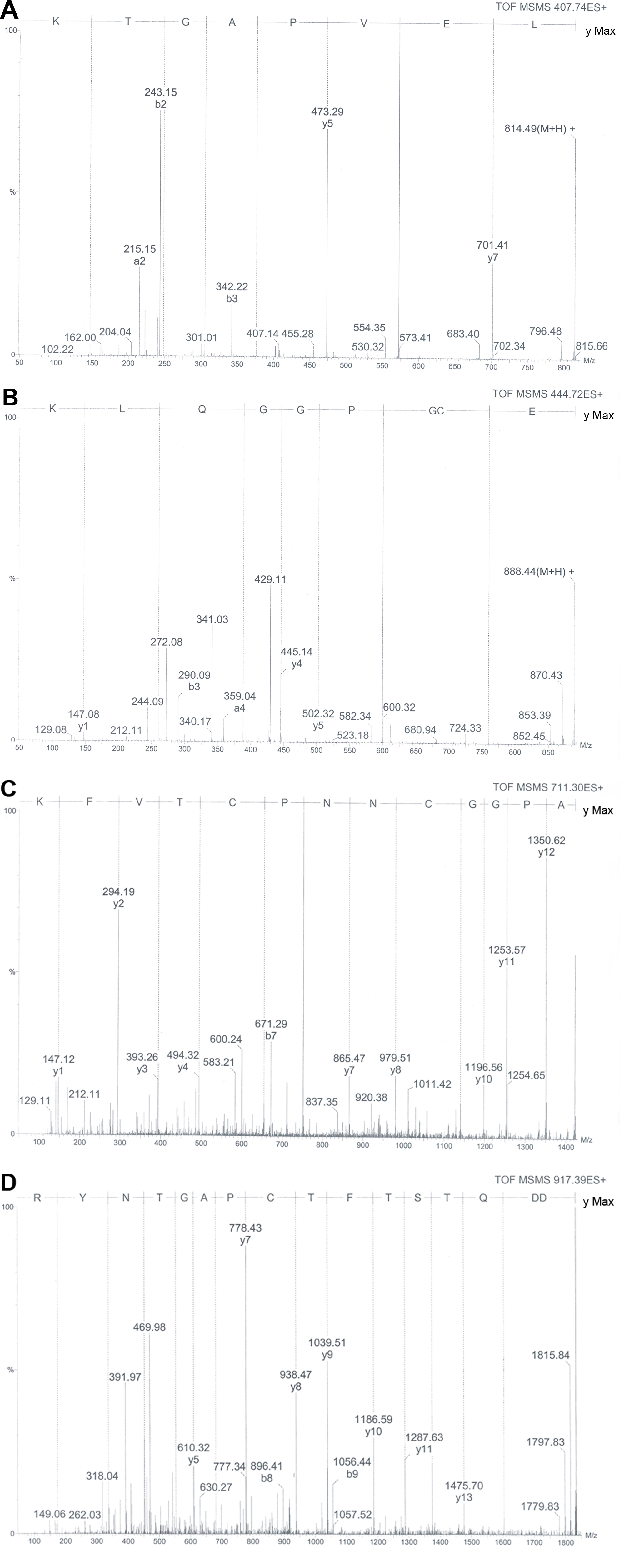

Supplement: Figure S1 — nanoESI-MS/MS spectrums analysis of four polypeptide fragments from CkTLP protein. A. nanoESI-MS/MS spectrum of the [M+H] 2+ ion (m/z 407.74). The mass differences of the consecutive yn ions: m/z 814.49(y8), m/z 701.41(y7), m/z 572.36 (y6), m/z 473.29 (y5), m/z 376.24(y4), m/z 305.20 (y3), m/z 248.17 (y2) and m/z 147.24 (y1) and their correspondence to the amino acid sequence at the top of spectrum are shown. B. nanoESI-MS/MS spectrum of the [M+H] 2+ ion (m/z 444.72). The mass difference of the consecutive yn ions: m/z 888.44(y9), m/z 759.40(y8), m/z 656.37 (y7), m/z 599.37 (y6), m/z 502.32 (y5), m/z 445.14(y4), m/z 388.28 (y3), m/z 260.21 (y2) and m/z 147.08 (y1) and their correspondence to the amino acid sequence at the top of spectrum are shown. C. nanoESI-MS/MS spectrum of the [M+H] 2+ ion (m/z 711.30). The mass difference of the consecutive yn ions: m/z 1421.66(y13), m/z 1350.62 (y12), m/z 1253.57 (y11), m/z 1196.56(y10), m/z 1139.53(y9), m/z 979.51 (y8), m/z 865.47 (y7), m/z 751.41 (y6), m/z 654.34 (y5), m/z 494.32(y4), m/z 393.26 (y3), m/z 294.19 (y2) and m/z 147.12 (y1) and their correspondence to the amino acid sequence at the top of spectrum are shown. D. nanoESI-MS/MS spectrum of the [M+H] 2+ ion (m/z 917.39). The mass difference of the consecutive yn ions: m/z 1833.78 (y16), m/z 1718.76 (y15), m/z 1603.76(y14), m/z 1475.70 (y13), m/z 1374.68(y12), m/z 1287.63 (y11), m/z 1186.59 (y10), m/z 1039.51 (y9), m/z 938.47 (y8), m/z 778.43 (y7), m/z 681.41 (y6), m/z 610.32 (y5), m/z 553.31(y4), m/z 452.25 (y3), m/z 338.31 (y2) and m/z 175.13(y1) and their correspondence to the amino acid sequence at the top of spectrum are shown. (TIF) [file pone.0016930.s001.tif]

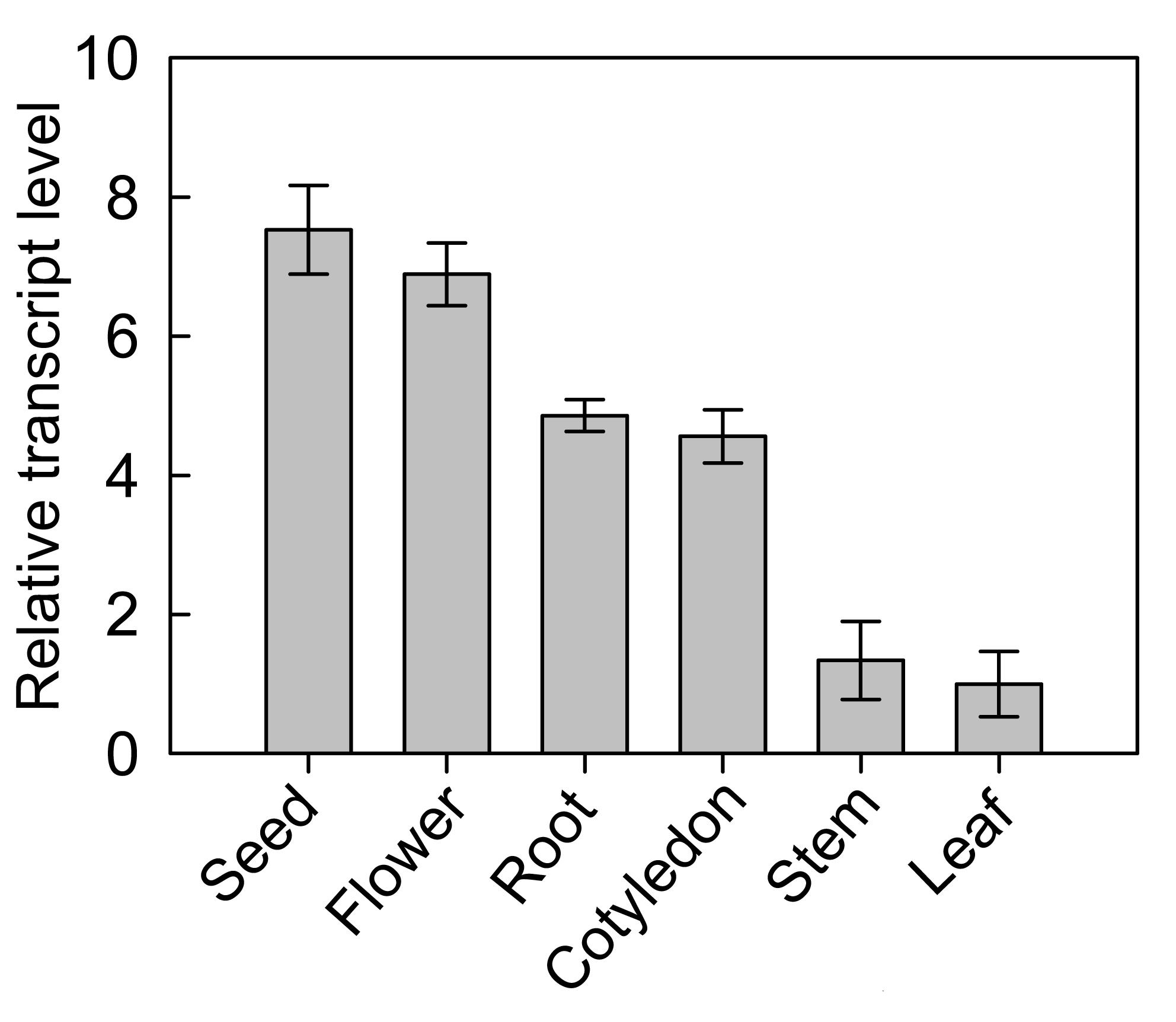

Supplement: Figure S2 — Real-time PCR analysis of CkTLP relative transcript level in different tissues of C. komarovii . Total RNA were extracted from roots, stems, leaves of 3-week-old plants, cotyledons of 1-week-old seedlings and mature seeds. The CT values of CkTLP obtained from real-time PCR were normalized against those of EF1-α (yielding ΔCT±SD with three biological and three technical replicates). The relative transcript level of CkTLP was calculated using the formula Xfold = 2−ΔΔCT with leaf used as reference condition. Date represent mean±SD (n = 3). (TIF) [file pone.0016930.s002.tif]
